# Association Between *Toxoplasma gondii* Infection and Serum Neurotransmitter Levels in Major Depressive Disorder Patients: A Case-Control Study in Bangladesh

**DOI:** 10.1155/japr/7054920

**Published:** 2024-12-19

**Authors:** Jerin E. Gulshan, Samia Sultana Lira, M. M. A. Shalahuddin Qusar, Md. Ismail Hosen, Atiqur Rahman, Md. Rabiul Islam, Taibur Rahman

**Affiliations:** ^1^Laboratory of Infection Biology, Department of Biochemistry and Molecular Biology, University of Dhaka, Dhaka 1000, Bangladesh; ^2^Department of Psychiatry, Bangabandhu Sheikh Mujib Medical University, Dhaka 1000, Bangladesh; ^3^Laboratory of Clinical Biochemistry and Translational Medicine, Department of Biochemistry and Molecular Biology, University of Dhaka, Dhaka 1000, Bangladesh; ^4^School of Pharmacy, BRAC University, Dhaka 1212, Bangladesh

**Keywords:** adrenaline, dopamine, major depression, noradrenaline, *Toxoplasma gondii*

## Abstract

*Toxoplasma gondii* (*T. gondii*) is an obligate, intracellular, neurotropic protozoan parasite. After primary infection, *T. gondii* parasite undergoes stage conversion from fast-replicating tachyzoites to slow-replicating dormant bradyzoites, particularly in the brain, and persists for a lifetime of an individual. In this study, the impact of *T. gondii* infection in individuals with psychological disorder, that is, major depressive disorder (MDD) has been studied. Ninety-five MDD (*n* = 95) patients were enrolled with age and sex-matched healthy controls (HCs, *n* = 90). The seroprevalence of *T. gondii* infection among these individuals was determined using the TOXO IgM/IgG Rapid Test Cassette that determines the anti-*T. gondii* IgM and IgG antibodies in the serum samples. Furthermore, to understand the impact of *T. gondii* in developing major depression, the serum level of neurotransmitters (i.e., dopamine, adrenaline, and noradrenaline) was determined using an enzyme-linked immunosorbent assay (ELISA). Our data suggest that anti-*T. gondii* IgG was slightly higher in MDD patients than in HCs. The level of dopamine was significantly lower in *T. gondii*-infected MDD patients than in HCs. However, adrenaline and noradrenaline levels showed increasing levels in *T. gondii*-infected MDD patients. The level of neurotransmitters was correlated with the DSM-D scores of MDD patients. These data, nevertheless, confirm that *T. gondii* might affect the level of neurotransmitters in MDD patients. However, whether the reduced level of dopamine and increased level of adrenaline and noradrenaline act as contributing factors for the development of MDD is yet to be known.

## 1. Introduction

*Toxoplasma gondii* is an apicomplexan zoonotic neurotropic protozoan parasite that causes toxoplasmosis and infects 30%–50% of the total world population. Being discovered in 1908, this, as one of the most widespread parasites, has given rise to infrequent outbreaks and epidemics in the last century. It has an unusually wide range of intermediate hosts encompassing warm-blooded animals, while felids are its strict definitive hosts [1–9]. Potential media for infection into humans include consumption of raw or inadequately cooked meat of infected livestock animals, oocyst-contaminated vegetables, foods, and water and exposure to contaminated soil [10, 11].

Felids (such as cats) shed environmentally resistant oocysts after taking up any of the three infectious stages: (1) rapidly dividing invasive tachyzoites, (2) slowly dividing bradyzoites, and (3) environmentally stable encysted sporozoites [12]. Upon ingestion by intermediate hosts (such as humans), the sporozoites get freed from oocyst by proteolytic enzymatic activity in the host stomach and small intestine. Sporozoites then convert into tachyzoites which disseminate in all organs through systemic circulation. Depending on the susceptibility and immune status of hosts, the acute infection can vary in severity. Under normal conditions, the host immune response can effectively take control over the infection. However, the tachyzoites rapidly differentiate into cyst-enclosed bradyzoites. These latent cells can persist in the host's long-lived cells, predominantly in neural and muscle tissues throughout the host's lifetime [13–16]. Although the current drug therapy works against the tachyzoite stage, existing treatment cannot clear out the bradyzoite stage [3]. The quiescent bradyzoites in the cyst, upon immune suppression, can revert into proliferating tachyzoites leading to devastating tissue destruction [17]. *T. gondii* infection has been attributed to severe toxoplasmosis in case of immunosuppressed persons and after transfusions or transplantations [18–26]. Moreover, congenital toxoplasmosis can cause a wide variety of manifestations in fetus and infant such as stillbirth and spontaneous abortion in human and animals, thereby affecting livestock production [27–29].

Although primary *T. gondii* infection is mostly asymptomatic, tissue cysts containing bradyzoites are suspected to cause neuronal cell death that could ultimately result in neurological impairment [30]. In several cases, chronic infection has been associated with altered behavior and mental disorders like personality changes, schizophrenia, suicidal tendencies, bipolar disorder, and depression in humans [31–38]. Studies on mouse models concluded that the parasite poses a great impact on rodent behavior [39–41]. On top of that, the magnitude of behavior alteration has been found to be directly associated with the cyst burden in the brain [42].

Major depressive disorder (MDD) is considered one of the global burdens of chronic illness. Globally, more than 264 million people suffer from depression, and the contribution of MDD to total mortality accounts for 10%. MDD is characterized by mood changes, diminished interests, deteriorated pleasure and cognitive function, and disturbed appetite or sleep. The underlying reasons for developing MDD are thought to be genetic, psychological, environmental, family history of psychological disorders, medication on certain drugs, history of trauma or abuse, etc. [43–45]. While also having the opposite possibility [46, 47], there exists evidence that *T. gondii* infection has the potential to cause depression. For instance, the seroprevalence of *T. gondii* infection was reported to be greater in depressed patients relative to a control group. In addition, one patient who was previously unresponsive to conventional antidepressants has recovered from depression following treatment for an underlying *T. gondii* infection [30, 48]. We hypothesized that *T. gondii* chronic infection is associated with the development of MDD since the tissue cysts can affect the neuromodulatory system and neurotransmission.

**Figure 1:**
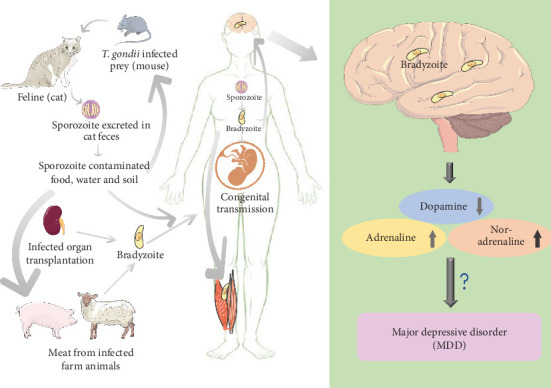
Proposed model of *T. gondii*-mediated changes on neurotransmitter level.

Neurotransmitters, which are chemical messengers essential for facilitating communication between neurons within the synapses of the nervous system, play a crucial role in the transmission of information from the presynaptic neuron to the postsynaptic neuron. This ensures that the stimulus reaches the appropriate location at the appropriate time in the corresponding region of the reward system. Dopamine, adrenaline, and noradrenaline are three monoamine neurotransmitters associated with depression (Figure 1). Based on evidence of reduced norepinephrine metabolism, elevated tyrosine hydroxylase activity, and decreased density of norepinephrine transporter in the locus coeruleus in depressed patients, it has been hypothesized that central noradrenergic system dysfunction contributes to the pathophysiology of MDD.

To address the hypothesis, this study was designed to evaluate the association between *T. gondii* infection and MDD, furthermore, assessing the effect of toxoplasmosis infection on the level of dopamine, adrenaline, and noradrenaline.

## 2. Material and Methods

### 2.1. Study Design and Subject Enrollment

Ninety-five MDD patients attending the department of Psychiatry, Bangabandhu Sheikh Mujib Medical University (BSMMU), Dhaka, Bangladesh, and 90 sex and age-matched healthy controls (HCs) (write how to choose them and from where). The enrolled subjects were interviewed according to the American Psychiatric Association's Diagnostic and Statistical Manual of Mental Disorder, fifth edition (DSM-V) using a predesigned questionnaire for recordation of their sociodemographic data. According to the manual, subjects with DSM-score > 3 were considered as MDD patients. We applied DSM- D rating depression. Among the MDD patients for measuring the degree of depression [49], scores 3–6 were considered as moderately depressed, and scores 7–9 were considered as major depressed.

### 2.2. Serum Collection

Venous blood was collected from the study subjects using standard blood collection apparatus. After collection into 5 mL red top tubes containing clot activators (ASPO Medical Equipment Co. Ltd), the whole blood was allowed to clot by leaving it undisturbed for 15–30 min at room temperature. After centrifugation at 1000 × g for 15 min, the resultant supernatant (serum) was transferred immediately into a microcentrifuge tube [50]. The samples were stored at −20°C, and 2°C–8°C were maintained while handling.

### 2.3. Detection of Anti-*T. gondii* IgM and IgG in Serum

*T. gondii* infection was confirmed by using the TOXO IgM/IgG Rapid Test Cassette (Cortez Diagnostics, United States) in the serum of MDD patients and HCs. Upon formation or absence of colored lines, four types of specimens were detected—(1) IgG positive, (2) IgM positive, (3) negative, and (4) invalid. The assay result was read in 15 min. No result was interpreted after 20 min.

### 2.4. Measurement of Dopamine, Adrenaline, and Noradrenaline in Serum Using Enzyme-Linked Immunosorbent Assay (ELISA)

For measurement of dopamine, adrenaline, and noradrenaline levels in serum, ELISA was performed. Manufacturer guideline of 3-CAT ELISA Kit (LDN, Germany) was followed for extraction of neurotransmitters from serum and subsequent measurement by ELISA.

### 2.5. Extraction of Adrenaline, Noradrenaline, and Dopamine From Serum

The three catecholamines, adrenaline, noradrenaline, and dopamine were extracted following the extraction procedure of cis-diol specific affinity gel which is converted enzymatically followed by acylation. A microtiter plate format is used in this ELISA kit where the antigen was bound to the solid phase of the plate. The system was allowed to reach equilibrium and free antigen; free antigen-antibody complexes were removed by washing. An anti-rabbit IgG-peroxidase conjugate was used to detect the antibody bound to the solid phase, and the reaction was monitored at 450 nm. Unknown samples were quantified by comparing their absorbance with a preset standard curve with known concentrations. Repeated freezing and thawing of reagents and specimens were recommended to avoid. Only deionized, distilled, or ultrapure water was used for dilution or reconstitution purposes, and all steps were completed without interruption. One thousand milliliters of water was used to dilute the 20-mL wash buffer. Reconstitution of enzyme was done by 1 mL of water, and 0.3 mL of coenzyme was added followed by 0.7 mL of adjustment buffer which makes the total volume 2 mL. For sample preparation, 10 *μ*L of standards, controls, and 300 *μ*L of serum samples were pipetted into the respective wells of the extraction plate. Two hundred and fifty microliters of water and 50 *μ*l of assay buffer were added. After the addition of 50 *μ*l of extraction buffer, the plate was covered with adhesive foil and incubated for 35 min at room temperature (25°C) on a shaker (500 rpm). The foil was removed; the plate was blot dried by tapping it on absorbent material. One milliliter of wash buffer was added to all wells. The plate was incubated for 5 min at RT (20°C–25°C) on a shaker (500 rpm), emptied, and blot dried by tapping the inverted plate on absorbent material.

The plate was covered with adhesive foil and incubated for 10 min followed by the addition of 175 *μ*L HCl. Later, the foil was removed and discarded. To end, the supernatant was used for measuring adrenaline, noradrenaline, and dopamine by using ELISA assay.

### 2.6. Determination of the Level of Adrenaline, Noradrenaline, and Dopamine in Extracted Samples

Enzyme-linked immune sorbent assay was used to determine the level of adrenaline, noradrenaline, and dopamine in extracted samples. Firstly, 25 *μ*L of the respective enzyme solution and then 100, 20, and 50 *μ*L of extracted standards, controls, and samples for adrenaline, noradrenaline, and dopamine, respectively, were added into the wells. Incubation was done for 35 min at room temperature on a shaker. Antiserum of the respective adrenaline, noradrenaline, and dopamine was added, covered with adhesive foil, and incubated for 2 h on a shaker. The foil was removed, and the content of the wells was discarded or aspirated. The plate was washed thrice by adding 300 *μ*L of wash buffer, and the content was discarded and blotted dry each time by tapping the inverted plate on absorbent material and further incubated for 30 min on a shaker after adding 100 *μ*L of enzyme conjugate. The contents of the wells were aspirated, the plate was washed thrice by adding 300 *μ*L of wash buffer, and the content was discarded and blotted dry each time by tapping the inverted plate on absorbent material. One hundred microliters of the substrate was added into the wells and incubated for 25 min at room temperature. Caution was taken at this step to avoid exposure to sunlight, 100 *μ*L stop solution was added to each well, and the microtiter plate was shaken to ensure a homogenous distribution of the solution. The absorbance was taken within 10 min of the experiment using a microplate reader set to 450 nm (Stat Fax 4200, Awareness Technology Inc, United States).

### 2.7. Statistical Analysis

Data was tabulated and analyzed using GraphPad Prism Version 9.0. Quantitative data is expressed by numbers and percentages, qualitative data is expressed by mean ± SEM (standard error mean). Statistically significant differences were calculated and considered significant if *p* < 0.05.

## 3. Results

### 3.1. Sociodemographic Characteristics

**Figure 2:**
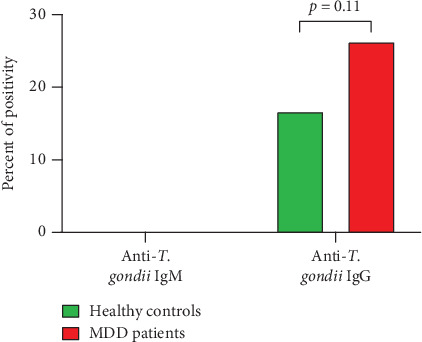
The rate of seropositivity of *T. gondii* in MDD is higher than in healthy controls. The percentage of anti-*T. gondii* IgG-positive MDD cases and healthy controls were calculated and presented. *p* value was calculated using chi-square test between healthy controls and MDD cases and considered significant if *p* < 0.05.

**Figure 3:**
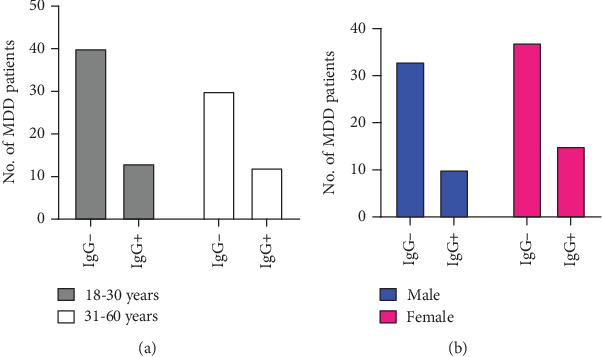
Comparison of seropositivity of *T. gondii* in MDD patients with age groups and genders. (a) The anti-*T. gondii* IgG positive and negative cases for age groups 18–30 and 31–60 years were calculated and expressed as the absolute number. (b) In addition, the anti-*T. gondii* IgG-positive and negative cases were compared for both male and female groups and expressed as the absolute number.

The sociodemographic characteristics and distribution of *T. gondii* infection of MDD patients are shown in Table 1. The seroprevalence of *T. gondii* was found to be higher in MDD patients compared to HCs. Out of 95 MDD patients, 25 were found to be seropositive for anti*-T. gondii* IgG antibodies, whereas 70 were seronegative. In the case of HCs (*n* = 90), the numbers of seropositive and seronegative for anti*-T. gondii* IgG antibodies were 15 and 76, respectively. MDD patients had a higher rate of seropositivity for anti*-T. gondii* IgG antibodies (26.3%) than that of HCs (16.7%) (Figure 2). However, no anti*-T. gondii* IgM antibody was found in the serum of either study group. *T. gondii* infection was found to be different among sex and age groups. While the infection rate being slightly higher in female (28.84%) than in male (23.25%), older subjects had an increased rate of *T. gondii* infection (28.57%) compared to younger ones (24.52%) (Table 1) (Figure 3).

Data of *T. gondii* infection does not show any correlation with the socioeconomic status, smoking habit, and BMI of MDD patients (data not shown). There is a linear relationship between the total number of MDD subjects in all three smoking habit groups (ex-smoker (77), current smoker (20), and nonsmoker (52)) and seroprevalence rate for the respective groups (ex-smoker (27%), current smoker (20%), and nonsmoker (27%)) (Table 1). The parasite infection does not show any association with the BMI; the proportion for underweight, normal, and overweight subjects were determined to be 25%, 28.76%, and 14.28%, respectively (Table 1). Out of 95 MDD patients, 23 had a family history of MDD (24.21%); 9 of which (39.13%) were found to be positive for *T. gondii* IgG antibody. On the other hand, 72 subjects were not reported to have MDD in their family, but 16 of them (22.22%) were seropositive to the parasite antibody (Table 1). Data also show that the prevalence of MDD and *T. gondii* infection are found to be higher in the rural and suburban areas and among less-educated subjects.

### 3.2. *T. gondii* and the Level of Adrenaline in MDD Patients

**Figure 4:**
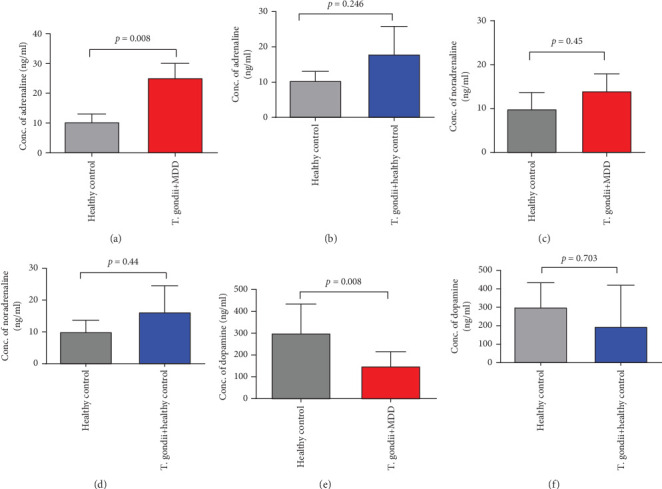
Level of adrenaline, noradrenaline, and dopamine in the serum of MDD patients and healthy controls. The study subjects were divided into three groups: (a) healthy control, *n* = 24; (b) *T. gondii-*positive MDD patients, *n* = 24; (c) *T. gondii* positive healthy control, *n* = 8. Concentration (nanograms per milliliter) of adrenaline, noradrenaline, and dopamine was measured through Enzyme-Linked Immunosorbent Assay (ELISA) in the serum of the abovementioned groups. Here, a comparison of the level of (a) adrenalin, (b) noradrenaline, and (c) dopamine was shown between healthy control and *T. gondii*-positive MDD patients. Besides, the impact of *T. gondii* was further confirmed by comparing the level of (d) adrenaline, (e) noradrenaline, and (f) dopamine between healthy control and *T. gondii*-positive healthy control. *X*-axis represents sample population groups, and *Y*-axis represents the concentration of respective neurotransmitters in nanograms per milliliter. Data were expressed as mean ± SEM (standard error mean). Significant differences were calculated by unpaired students t-test and considered significant if *p* < 0.05.

We found a significantly higher level of adrenaline in the serum of *T. gondii* IgG-positive MDD patients as compared to HC individuals (Figure 4(a)). This suggests that chronic *T. gondii* infection may have a role in the level of adrenaline in MDD patients. To check the impact of the parasite on adrenaline levels, we tried to find out the level of adrenaline in Toxo-negative and Toxo-positive HC individuals. Results showed that adrenaline level was higher in Toxo-positive HC individuals as compared to Toxo-negative HC individuals (Figure 4(b)).

### 3.3. *T. gondii* and the Levels of Noradrenaline in Serum of MDD Patients

We found a slightly increased level of noradrenaline in the group of Toxo-positive MDD cases compared to HC individuals (Figure 4(c)). Similarly, we found a slightly higher level of noradrenaline in Toxo-positive HC compared to Toxo-negative HC (Figure 4(d)).

### 3.4. *T. gondii* and Dopamine Level in Serum of MDD Patients

In our study, the level of dopamine was measured in Toxo-positive MDD cases and HCs. We found a significantly lower level of dopamine in Tox-positive MDD patients as compared to HC individuals (Figure 4(e)). To further confirm, we checked dopamine levels in the serum of Toxo-negative and Toxo-positive HC individuals and found a similar trend (Figure 4(f)).

### 3.5. Level of Adrenaline, Noradrenaline, and Dopamine and the DSM-Score of MDD Patients

**Figure 5:**
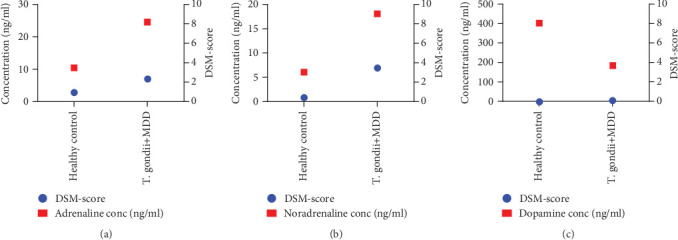
Correlation of the concentration of adrenaline, noradrenaline, and dopamine with DSM-score. In this figure, the concentration of (a) adrenaline, (b) noradrenaline, and (c) dopamine (nanograms per milliliter) was correlated with the respective depressive index calculation (DSM) score. The concentration was calculated as the mean for the healthy control and *T. gondii*-positive MDD samples and plotted with the DSM score obtained during patients' interviews to diagnose major depression. The *X*-axis shows sample groups, the left *Y*-axis represents concentration (nanograms per milliliter), and the right *Y*-axis represents the DSM score. GraphPad Prism Version 9.0 was used to prepare the figure.

It was found that the average concentration of serum adrenaline is directly proportional to the DSM-Score of the MDD patients (Figure 5(a)). This suggests that the increased level of adrenaline is associated with the clinical symptoms of MDD. It was found that the average concentration of serum noradrenaline was directly proportional to the DSM-score of the MDD patients (Figure 5(b)).

## 4. Discussion

This retrospective study conducted on 95 MDD patients and 90 HC individuals was mainly concerned to determine the prevalence of *T. gondii* infection in the Bangladeshi population and its association with the development of MDD. One of the major findings of the study is that *T. gondii*-infected MDD patients have shown significantly higher levels of adrenaline and lower levels of dopamine in comparison to healthy individuals.

The seroprevalence of *T. gondii* differs depending on the environment, socioeconomic condition, the general level of hygiene, culture, food preparation practice, eating habits, etc. [51, 52]. According to a cross-sectional analysis conducted in the United States, the risk for *T. gondii* infection increases with age and is higher among lowly educated people and the ones involved in soil-related occupations [53]. Our data show consistency with the findings in the mentioned study.

In the perspective of Bangladesh, household works including cooking are mainly handled by women. So, they are more susceptible to exposure to parasite-contaminated raw meat. The seroprevalence of *T. gondii* infection is a little higher in the age group 18–30 years. This can be supported by the higher consumption of meat and elevated exposure of *T. gondii* oocysts by this group.

Besides, lack of proper knowledge about personal hygiene and resultant lower quality of health services and hygienic standards can be a possible explanation for higher seroprevalence in poorly educated population. Correlation with socioeconomic status in our sample is not inferential because samples are not evenly distributed for different economic categories. In the social context of Bangladesh, smoking is almost invariably limited to males. Although women had a higher rate of infection, there were not any female smoker in the study subjects. The proportional relationship between the total cases of MDD and the seroprevalence regarding smoking habits infers that there is no significant impact of smoking on either MDD development or *T. gondii* infection. The same goes for BMI indicating that MDD or *T. gondii* infection takes place irrespective of BMI of an individual.

In the present study, we identified that subjects living in rural and suburban areas have a higher rate of infection. Greater exposure to land soil in people living in rural and suburban areas can be a possible cause for this susceptibility. In a European case-control study, exposure to soil was found to be a strong risk factor causing 6%–17% of primary infection in humans [54]. Contact with soil was reported as a major factor in another study conducted on 2126 pregnant women in Brazil [55].

Humid climate and agrarian economy in Bangladesh are appropriate for *T. gondii* transmission because moist soil is ideal for oocysts where they can survive for months to years. Domestic cats tend to bury their feces in soft and moist soil grass, grain, or streets. Not only that, pastoral domestic animals such as sheep, goats, and chickens can be infected by ingesting contaminated soil while grazing. However, research is insufficient about the survival rate of oocyst in sun exposure. In Texas (6°C–36°C), oocyst survived in cat feces for 334 days when covered and 46 days when it was uncovered. Oocysts get killed by temperatures above 60°C [6, 56–58]. The geographical position of Bangladesh also poses a threat for *T gondii* infection since the low altitude is reported as a risk factor [59]. Remarkably, all the seropositive cases tested positive for IgG antibody only, not IgM. This implies that the parasite by chronically persisting in the body for a long time could get the chance to alter brain tissue morphology and functionality.

Dysregulation of catecholamine (e.g., adrenaline, noradrenaline, and dopamine) in the aetiopathophysiology of depression is well documented [60–63]. We found an increase in adrenaline and noradrenaline levels in IgG-positive MDD patients than in healthy subjects. Since downregulation of these neurotransmitters has been observed in depression in other studies, the employment of more robust techniques is required to interpret these findings [64–66]. Previous studies have reported a reduction of dopamine release in depressive disorders [61–66]. The data obtained in our study is consistent with this finding. Interestingly, dopamine is hypothesized to increase in *T. gondii* infection [67, 68]. *T. gondii* possesses genes for tyrosine hydroxylase that are directly involved in and thereby could potentially influence dopamine and serotonin biosynthesis. Furthermore, L-DOPA production is reported to be induced during bradyzoite production [69]. A study by Stibbs et al. in rodent models shows significant neurochemical changes during *T. gondii* acute infection, such as a 40% rise in homovanillic acid and a reduction in noradrenaline level. Although dopamine levels were not altered during acute infection, an increased level was found during chronic infections in mice. However, serotonin levels remained unchanged [70, 71]. In an investigation, researchers from the University of Leeds found an increase in dopamine metabolism in *T. gondii*-infected neural cells, while the presence of tyrosine hydroxylase was prevalent in intracellular tissue cysts [68]. Moreover, tryptophan starvation being a key path of host defense against *T. gondii* proliferation [72], inflammatory cytokine-mediated activation of indoleamine 2,3-dioxygenase (IDO) may cause detrimental behavioral changes including depression [73]. Thereby, the contribution of *T. gondii* infection in MDD development through dopamine level change remains elusive. Taken together, these findings strengthen our hypothesis that there might be an association between *T. gondii* seroprevalence and the development of neurological disorders like MDD. Our findings also raise the possibility of the existence of a third factor such as hygienic or eating habits which could influence both the *T. gondii* seroprevalence and occurrence of MDD.

Although these results represent an impactful initial step towards further understanding the topic, our study is subject to some limitations. Firstly, some medical histories of the patients and HCs, such as duration, persistence, and type of antipsychotic medication administered, have not been taken into consideration. These factors, having the potential to greatly influence the study results such as DSM-V scores, can limit our perception along with the conclusion of the correlation study. Secondly, we used a retrospective study because of time and resource efficiency. Not being a longitudinal study, it has the possibility of missing out on some incites which are visible only when observed for a longer period. Thirdly, mental health is an issue that is given less attention in the societies of Bangladesh. This situation gives rise to the possibility of not getting the whole scenario as a result of MDD being less reported. Lastly, samples were collected from only one city in Bangladesh and not evenly distributed for categories like socioeconomic conditions. These factors regarding sampling can lead to the absence of some valuable points of view. The strength of our study is that we employed techniques to examine the neuromodulation in neurotransmitter levels not just qualitatively, but also quantitatively. Future investigation with multisource clustered sampling and adopting more advanced techniques can provide a comprehensive idea and stronger evidence to inspect our theory.

## 5. Conclusion

MDD is a complex neurological disorder; therefore, it is hard to pinpoint one single factor responsible for its occurrence. From our study, it can be concluded that chronic *T. gondii* infection is present in one out of four MDD patients in Bangladesh. Additionally, the direct or indirect role of *T. gondii* was observed in the reduced level of dopamine and slight increase in adrenaline and noradrenaline levels; these can play a crucial role in the development of MDD. Further studies are warranted to establish the mechanism by which T*. gondii* influences neurotransmission and the development of MDD.

## Figures and Tables

**Table 1:** Sociodemographic characteristics of major depressive disorder patients.

**Parameter**	**No of cases**	**IgG (+)ve**		**IgG (−)ve**	
		**No**	**Proportion (%)**	**No**	**Proportion (%)**
Gender					
Male	43	10	23.25	33	76.74
Female	52	15	28.84	37	71.15
Age (years)					
18–30	53	13	24.52	40	75.47
31–60	42	12	28.57	30	71.43
Economic status					
Poor	26	8	30.71	18	69.23
Lower-middle class	43	12	27.9	2	4.65
Middle class	3	0	0	3	100
Upper-middle class	15	2	13.33	13	86.67
Rich	8	3	37.5	5	62.5
Family history of psychological disorder					
Yes	23	9	39.13	14	60.87
No	72	16	22.22	56	77.78
Previous history of psychological disorder					
Yes	58	16	27.5	42	72.41
No	37	9	24.32	28	75.68
Smoking habit					
Nonsmoker	70	19	27.14	67	95.71
Current smoker	20	4	20	0	0
Ex-smoker	52	14	26.92	3	5.77
Education					
Primary	16	8	50	8	50
Secondary	26	5	19.23	21	80.77
Higher secondary	27	7	25.92	20	74.07
Graduate	26	5	19.23	21	80.77
Area of residence					
Urban	38	9	23.68	29	76.32
Suburban	13	4	30.77	9	69.23
Rural	44	12	27.27	32	72.72
BMI score					
Underweight	8	2	25	6	75
Normal	73	21	28.7	52	71.23
Overweight	14	2	14.28	12	85.71
DSM-V score					
3–6	34	6	17.6	28	82.35
7–9	61	19	31.14	42	68.85

## Data Availability

The datasets supporting the results and conclusion of this article were included within the manuscript file. However, the original dataset can be given upon request.
